# Asthma Is More Severe in Older Adults

**DOI:** 10.1371/journal.pone.0133490

**Published:** 2015-07-22

**Authors:** Joe G. Zein, Raed A. Dweik, Suzy A. Comhair, Eugene R. Bleecker, Wendy C. Moore, Stephen P. Peters, William W. Busse, Nizar N. Jarjour, William J. Calhoun, Mario Castro, K. Fan Chung, Anne Fitzpatrick, Elliot Israel, W. Gerald Teague, Sally E. Wenzel, Thomas E. Love, Benjamin M. Gaston, Serpil C. Erzurum, on behalf of The Severe Asthma Research Program

**Affiliations:** 1 Respiratory Institute, Cleveland Clinic, Cleveland, Ohio, United States of America; 2 Department of Pathobiology, Cleveland Clinic, Cleveland, Ohio, United States of America; 3 Center for Genomics and Personalized Medicine, Wake Forest University School of Medicine, Winston-Salem, North Carolina, United States of America; 4 Department of Medicine, The University of Wisconsin, School of Medicine and Public Health, Madison, Wisconsin, United States of America; 5 Department of Medicine, University of Texas Medical Branch, Galveston, Texas, United States of America; 6 Department of Medicine, Washington University School of Medicine, St. Louis, Missouri, United States of America; 7 The National Heart and Lung Institute, Imperial College, London, United Kingdom; 8 Department of Pediatrics, Emory University School of Medicine, Atlanta, Georgia, United States of America; 9 Pulmonary Division, Harvard Medical School, Brigham and Women’s Hospital, Boston, Massachussets, United States of America; 10 Department of Pediatrics, University of Virginia School of Medicine, Charlottesville, Virginia, United States of America; 11 Asthma Institute, The University of Pittsburgh, Pittsburgh, Pennsylvania, United States of America; 12 Department of Epidemiology and Biostatistics, Case Western Reserve University-MetroHealth Medical Center, Cleveland, Ohio, United States of America; 13 Department of Pediatric, Rainbow Babies and Children’s Hospital, Cleveland, Ohio, United States of America; University of Athens, GREECE

## Abstract

**Background:**

Severe asthma occurs more often in older adult patients. We hypothesized that the greater risk for severe asthma in older individuals is due to aging, and is independent of asthma duration.

**Methods:**

This is a cross-sectional study of prospectively collected data from adult participants (N=1130; 454 with severe asthma) enrolled from 2002 – 2011 in the Severe Asthma Research Program.

**Results:**

The association between age and the probability of severe asthma, which was performed by applying a Locally Weighted Scatterplot Smoother, revealed an inflection point at age 45 for risk of severe asthma. The probability of severe asthma increased with each year of life until 45 years and thereafter increased at a much slower rate. Asthma duration also increased the probability of severe asthma but had less effect than aging. After adjustment for most comorbidities of aging and for asthma duration using logistic regression, asthmatics older than 45 maintained the greater probability of severe asthma [OR: 2.73 (95 CI: 1.96; 3.81)]. After 45, the age-related risk of severe asthma continued to increase in men, but not in women.

**Conclusions:**

Overall, the impact of age and asthma duration on risk for asthma severity in men and women is greatest over times of 18-45 years of age; age has a greater effect than asthma duration on risk of severe asthma.

## Introduction

As the world is aging due to improved health care, new challenges emerge. Effective and efficient healthcare of the increasing numbers of older people require better understanding of the biology of aging, as well as the effect that aging has on severity of diseases in older individuals. This is particularly important in understanding lung health and disease in older individuals. While old age has been classically defined as age higher than 65 years, this arbitrary cutoff does not take into account the lung aging process that occurs throughout the course of life. After an initial rapid increase in the number of alveoli during the first 2 years of life, lung maturation slows down and achieves a relatively stable number of alveoli by age 8 [1, 2]. Lung size and airflow continues to increase until around the age of 20 in women and 25 in men. Following maturity, lung function declines throughout the remainder of life, with the rate of decline influenced by air pollution, cigarette smoke exposure, urbanization and climate changes [3–5]. Aging is also associated with increased stiffness of the chest wall, decreased elastic recoil of the lung and weaker respiratory muscles [6]. Small airways diameters start to decline after the fourth decade contributing to decreased expiratory flows seen with aging [7]. The loss of peri-bronchial lung tissue results in increasing closing capacity, and subsequent increased risk for collapse of small airways during tidal breathing [8–10]. Despite these known airway changes over the life-course, very little is known on the role of aging, as opposed to asthma duration, on the severity of asthma. The generalized decline in lung function might be expected to lead to more severe asthma in older patients [11, 12]. Cluster analyses performed to evaluate phenotypic diversity among adult asthmatics identified a unique phenotype of older asthmatics with severe symptoms and poor lung function [13–16]. Yet, these observations fail to differentiate whether the severity of asthma in older individuals is due to longer duration of chronic airway inflammation, or from the aging process on the asthmatic lung. We hypothesized that aging itself independent of asthma duration, contributes to the development of severe asthma.

## Methods

This is a cross-sectional study of a prospectively collected data. The analyses were conducted using data from adult participants (18 years and older) enrolled in the National Heart, Lung, and Blood Institute (NHLBI) Severe Asthma Research program (SARP) between 2002 to 2011. A written informed consent was obtained from all participants. The project and the informed consent were approved by the Institutional Review Board at all 10 SARP sites enrolling participants [17], which includes the Cleveland Clinic Institutional Review Board (IRB), Cleveland, OH; Case Western Reserve University IRB, Cleveland, OH; the IRB at Wake Forest University, Winston-Salem, NC; the IRB for health Sciences Research at the University of Virginia, Charlottesville, VA, the IRB at the University of Wisconsin, Madison, WI; University of Pittsburgh IRB, Pittsburgh, PA; The Human Research Protection Office (HRPO) at the Washington University, St. Louis, MO; Partners Human Research Committee at the Brigham and Women’s Hospital, Boston MA; the University of Texas Medical Branch IRB, Galveston, TX; Emory IRB, at Emory University School of Medicine, Atlanta, GA; and the Imperial College Research Ethics Committee (ICREC), at the National Heart and Lung Institute, Imperial College, London, UK. To be enrolled in the study, all adult participants provided a written informed consent. The informed consent as well as the study protocol were approved by all local IRBs. Children under the age of 18 years were excluded from this analysis.

Asthma severity (severe vs. non-severe asthma) was classified as defined by the proceedings of the American Thoracic Society Workshop on Refractory Asthma [18] and includes 2 major and 7 minor criteria. Asthma is considered to be severe if an individual meets one major and 2 minor criteria. Although a newer definition of severe asthma was published in 2014 [19], it was not in use when the data was prospectively collected between 2002–2011. Asthma related quality of life was assessed using the Asthma Quality of Life Questionnaire (AQLQ) [20], which consists of 32 questions in 4 domains (symptoms, activity limitation, emotional function and environmental stimuli). They address asthma-related symptoms and limitations during the preceding 2 weeks. Each item is scored on a scale of 1 (severely impaired) to 7 (no impairment).

### Statistical analyses

Patient characteristics, lung function, lab measurements were studied. Continuous normally distributed variables were reported using means and standard deviations (SD), and compared using Student T-test. Categorical variables were reported by their observed number and percent within the participant subsets, and compared using Pearson Chi-squared tests. Wilcoxon signed-rank test was used to compare all other variables. To test our hypothesis that asthma is worse in older individuals, and that it is independent of asthma duration, we performed a backwards selection by fitting a logistic regression model that sequentially removes the confounders with the largest p-value until all confounders in the model have a p-value < 0.10. The initial model included age of enrollment, asthma duration, gender, body mass index, race, history of gastro-esophageal reflux disease, history of hypertension, diabetes, coronary aretery disease, and diabetes, history of sinusitis and sinus surgery, history of nasal polyps and use of nasal corticosteroids, history of recurrent bronchitis, chronic bronchitis and pneumonia, history of allergies diagnosed by a physician and the number of positive skin reactions, passive and remote smoking history, family history of asthma, the presence of pets at home and whether symptoms are exacerbated by pets, peripheral blood percent eosinophil’s count and Immunoglobulin level, exhaled nitric oxide (eNO) or whether women where postmenopausal or using oral contraceptives or menopausal hormonal therapy. The final model included variables associated with severe asthma at a significance level of P less than 0.05. Such variables were age of enrollment, asthma duration, gastroesophageal reflux disease and the use of nasal corticosteroids. Prediction error of the model was assessed by measuring discrimination and Calibration. Discrimination was measured using of the c-statistic [21]. The C-index was 0.81. Calibration was measured using Le Cessie-van Houwelingen Normal Test Statistic (p = 0.68) [22].

The effect of age on treatment requirement and healthcare utilization was analyzed using a Propensity Score (PS) matching method with subsequent use of conditional logistic regression to adjust for gastroesophageal reflux disease (GERD) and asthma duration, which remained unbalanced after matching.

All above covariates assumed to affect asthma control were entered in a multivariate logistic regression model to determine the propensity of severe asthma (i.e., the conditional probability of severe asthma given a set of X covariates). Individual propensity scores were calculated without regard to outcomes. Individuals with missing covariates for the propensity score calculations were included. Continuous missing variables were imputed using the median value of such variable. Categorical missing variable were imputed at random. For propensity score matching, all 369 older asthmatics were matched with a 1:1 ratio without replacement with younger patients based on the logit of the propensity score using optimal pairwise propensity score matching. The logistic regression and propensity score matching were conducted using R Core Team (2014). R: A language and environment for statistical computing. R Foundation for Statistical Computing, Vienna, Austria

## Results

### Characteristics of study groups

**Fig 1:**
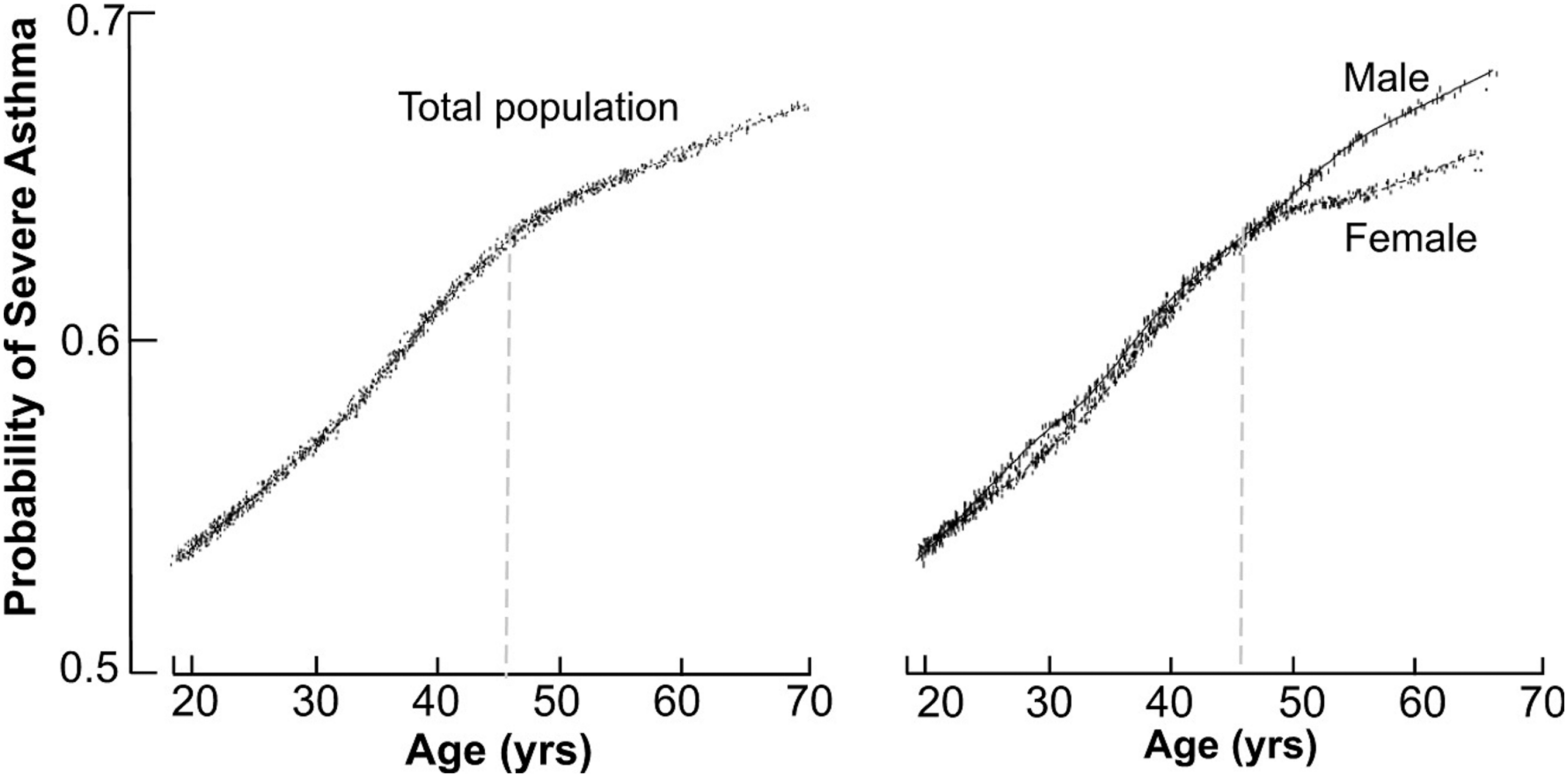
The probability of severe asthma as a function of age and stratified by gender. **[A]** The association between age and the probability of severe asthma by applying a LOWESS (Locally Weighted Scatterplot Smoother) smoother in the overall population. The relationship between age and probability of severe asthma resembles a spline with an inflection point at the age of 45 years. **[B]** The stratification by gender shows the probability of asthma severity is higher in men than women after age 45.

**Fig 2:**
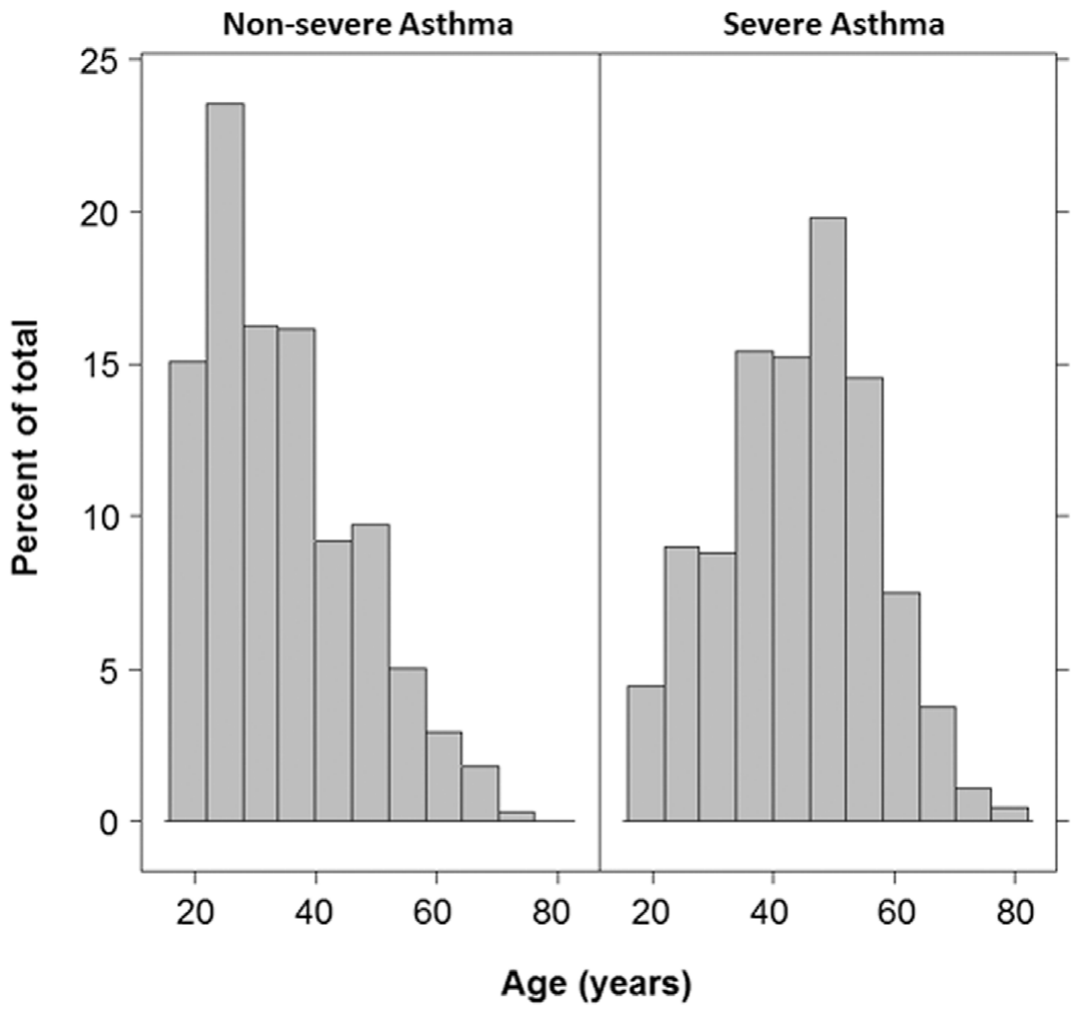
The histogram of age distribution by asthma severity. This histogram shows the difference in age distribution among severe and nonsevere asthmatics. The distribution of nonsevere asthmatics was skewed to the left reflecting younger age, and severe asthmatics were shifted to older age.

Initial analysis of the association between age of enrollment and the probability of severe asthma was performed by applying a Locally Weighted Scatterplot Smoother (LOWESS) to the 1130 adults in the SARP dataset [23]. This provides a visual snapshot of the relationship between those 2 variables by drawing a line through the central tendency of the data. The relationship between age and probability of severe asthma resembles a spline with an inflection point at the age of 45 years “Fig 1A” below which asthma severity increased rapidly as a function of age. After 45 the rise in asthma severity was slower. Visual inspection revealed a cutoff of 45years and the nonlinearity of the relationship between age and asthma severity was confirmed by comparing a logistic regression model of severe asthma as a linear function of age with a spline model using an inflection point at the age of 45. Accordingly, we grouped individuals into 2 groups, *i*.*e*. (1) older asthma (N = 369, 32.6%) who were older than 45 years, and (2) young adult asthma (N = 761, 67.4%), who were between ages 18–45 years. Older asthmatics were less likely to be African Americans or have atopy, but they reported more gastro-esophageal reflux disease, nasal polyps and history of sinusitis, and they used more inhaled corticosteroids than young adult asthma “Table 1”. They also had lower lung function, and higher airway neutrophilia suggested by bronchoalveolar lavage and induced sputum analysis “Table 1”. Gender stratification indicated that asthma severity is higher in both the older male and female asthmatics, but that the probability of severe asthma plateaued in women older than 45 years of age, *i*.*e*. increasing age in women over 45 did not result in increasing risk of severe asthma “Fig 1B”. Consistent with these findings, the age distribution of nonsevere asthmatics was skewed to the left reflecting younger age, and severe asthmatics were older “Fig 2”. The proportion of African American was similar among sevevere and nonsevere asthma.

**Table 1:** Characteristics of patients with asthma, according to age group.*

Characteristics		Young Adult Asthma (n = 761)	Older Asthma (n = 369)	p value†
**Age—yr**		30.5 ± 7.7	54.0 ± 7.0	<0.0001
**Sex—no (%)**				0.34
	**Male**	267 (35)	120 (32.5)	
	**Female**	494 (65)	249 (67.5)	
**Race—no (%)**				<0.0001
	**White**	466 (61)	274 (74)	
	**Black**	230 (30)	78 (21)	
	**Asian**	15 (2)	7 (2)	
	**Others**	50 (7)	10 (3)	
**BMI**		29.4 ± 8.3	31.0 ± 7.4	0.002
**Previous history of pneumonia—no (%)**		309 (41)	203 (55)	0.0003
**History of recurrent bronchitis—no (%)**		243 (32)	176 (48)	<0.0001
**History of sinusitis—no (%)**		280 (37)	197 (53)	<0.0001
**History of sinus surgery—no (%)**		84 (11)	82 (22)	<0.0001
**Nasal polyps—no (%)**		74 (10)	62 (17)	<0.0001
**History of nasal polyps removal—no (%)**		53 (7)	62 (17)	<0.0001
**GERD—no (%)**		165 (22)	154 (42)	<0.0001
**History of allergies—no (%)**		704 (93)	318 (86)	0.001
**Positive skin reactivity—no (%)**		667 (88)	282 (76)	<0.0001
**Symptoms caused by pets—no (%)**		549 (72)	208 (56)	<0.0001
**Symptoms caused by physical activity—no (%)**		480 (63)	282 (76)	<0.0001
**Number of positive skin reactions.**		4.36 (2.80)	3.22 (2.66)	<0.0001
**FEV1**				
	**Value—liters**	3.65 ± 1.52	2.25 ± 0.73	0.0009
	**Percent of predicted value**	92.11 ± 17.68	76.04 ± 20.08	<0.0001
**FVC**				
	**Value—liters**	4.2 ± 1.1	3.2 ± 0.9	<0.0001
	**Percent of predicted value**	99.4 ± 14.9	86.3 ± 16.5	<0.0001
**FEV1/FVC ratio**		0.72 ± 0.12	0.66 ± 0.12	<0.0001
**PC20 for methacholine—mg/ml**		2.5 ± 3.9	2.6 ± 4.5	0.6
**FENO—ppb**		37.2 ± 34.8	36.5 ± 34.4	0.73
**IgE—IU/m**		296.3 ± 631.8	260.4 ± 738.2	0.42
**Blood neutrophils— ×10** ^**−9**^ **/liter**		4.1 ± 2.0	4.5 ± 2.1	0.005
**Blood eosinophils— ×10** ^**−9**^ **/liter**		0.8 ± 0.5	0.3 ± 0.3	0.64
**Sputum Neutrophils–% of differential count**		40.1 ± 25.3	51.1 ± 27.0	<0.0001
**Sputum Eosinophils–% of differential count**		3.0 ± 7.8	3.7 ± 10.4	0.24
**Neutrophils in BAL–% of differential count**		1.9 ± 3.1	2.5 ± 4.5	0.03
**Eosinophils in BAL–% of differential count**		0.6 ± 1.3	0.7 ± 2.2	0.23

* Plus—minus values are means ±SD. BMI denotes body mass index, GERD gastroesophageal reflux disease, FEV1 forced expiratory volume in 1 second, FVC forced vital capacity, PC20 for methacholine the concentration of inhaled methacholine causing a 20% reduction in FEV1, FENO fraction of exhaled nitric oxide, and BAL broncho-alveolar lavage.

^†^ P values are for the comparison of older asthma with the young adult asthma group and were calculated with the use t-test for approximately normally distributed clinical characteristics, Pearson's chi-squared test for differences in proportions, and Wilcoxon signed rank test for all other variables.

### The role of age and asthma duration on asthma severity

**Fig 3:**
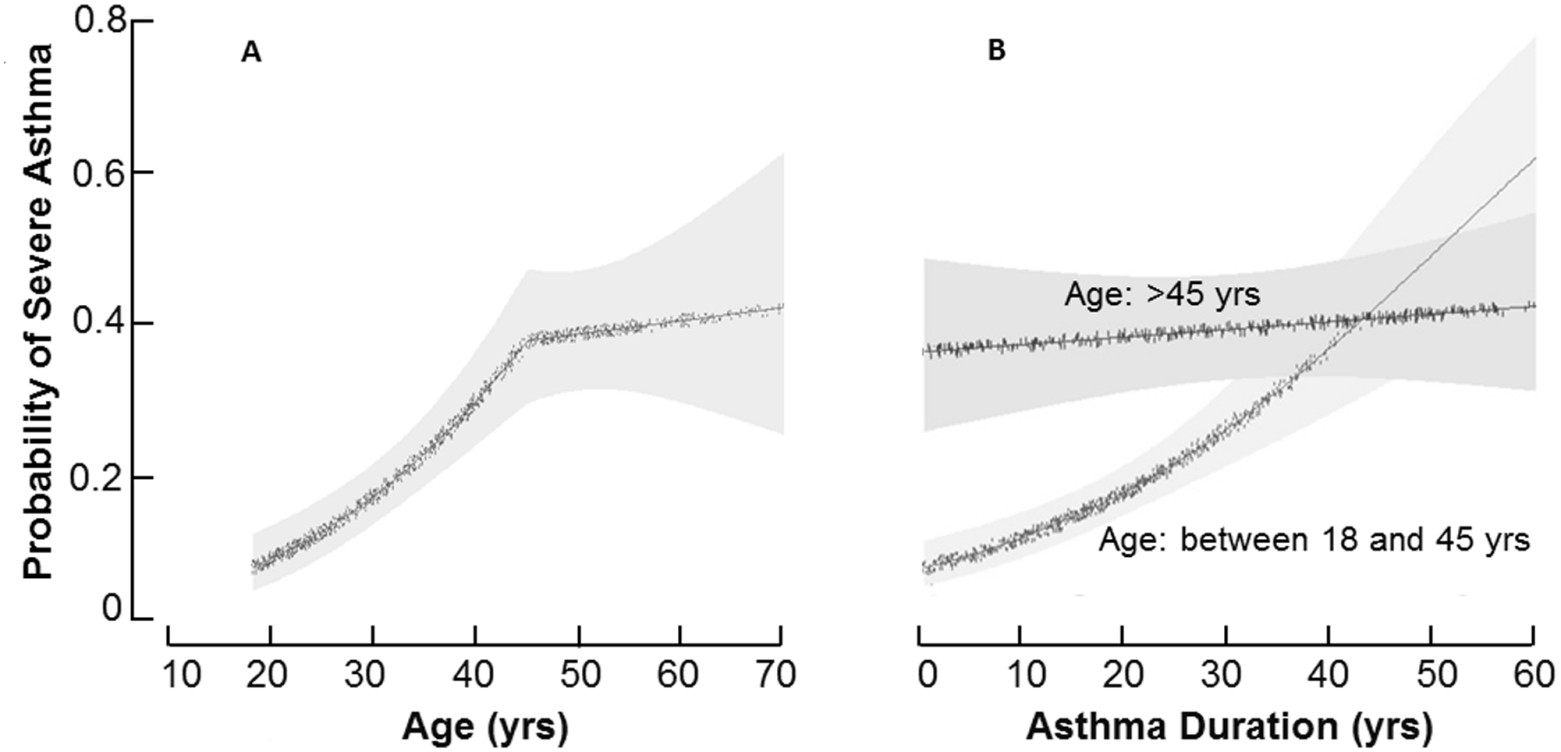
Probability of severe asthma as a function of age, and asthma duration. **[A]** The probability of severe asthma increases as a function of age until the age of 45 years, at which time the probability of severe asthma plateaus. The fitted final logistic regression model, which included variables associated with severe asthma and adjusted for asthma duration, a history of Gastro-esophageal Reflux Disease, and nasal corticosteroids use, was applied to calculate the probability of severe asthma. **[B]** Individuals were stratified by age groups into older or young adult asthma in order to evaluate the effect of asthma duration on risk of severe asthma in those older or younger than 45 years. Asthma duration has a lesser effect than age on the probability of severe asthma in younger adult asthmatics. In older asthmatics (age 45 years and older), asthma duration has no significant effect on risk of severe asthma.

Older asthmatics had a longer duration of asthma (30 ± 18 years) as compared to young adult asthmatics (19 ± 10 years) (p<0.01). Although asthma duration was greater in the older group, it was not meaningfully related to the age of the individual (R^2^ = 0.20, p<0.01). A fitted final logistic regression model, which included variables associated with severe asthma, was used to calculate the probability of severe asthma adjusting for asthma duration, as well as history of gastro-esophageal reflux disease and nasal corticosteroids use. Age continued to be significantly associated with risk of severe asthma until the age of 45 years [OR: 1.07(95 CI: 1.05; 1.09) per each year of age]. Beyond the age of 45 years, increasing age was no longer associated with severe asthma [OR: 1.01 (95 CI: 0.98; 1.04) per each year of age] “Fig 3A”.

Stratifying by age, the probability of severe asthma was clearly higher in older asthmatics as compared to the young adult asthmatics “Fig 3B”. The unadjusted analyses showed that older asthmatics have an odds ratio (OR) of 3.83 (95 CI: 2.95; 4.97) for severe asthma as compared to young adult asthma. Multivariate analysis showed that severe asthma is associated with age older than 45 [OR: 2.73 (95 CI: 1.96; 3.81)], history of Gastro-esophageal Reflux Disease [OR: 1.98(95 CI: 1.45; 2.70)], and the use of nasal corticosteroids [OR:4.6. (95 CI: 3.47; 6.18)] “Table 2”. All major and minor criteria of asthma severity [18] were more frequently seen with older age. Asthma severity was associated with longer asthma duration in the young adult asthmatic [OR: 1.04(95 CI:1.02;1.06) for each year increase in asthma duration]. However, asthma severity was not related to asthma duration in older asthmatics [OR: 1.00 (95 CI:0.99;1.02) per year].

**Table 2:** Logistic regression model of risk factors for severe asthma.*

	Unadjusted OR (95 CI)	Adjusted OR (95 CI)
**Age Category** †	3.83 (2.95; 4.97)	2.73 (1.96; 3.81)
**Asthma Duration** ‡	1.03 (1.02; 1.04)	1.02 (1.01; 1.03)
**Gastro-esophageal Reflux Disease**	2.82 (2.15; 3.70)	1.98 (1.45; 2.70)
**Use of Nasal Steroids**	4.99 (3.84, 6.48)	4.60 (3.47; 6.18)

* OR denotes odds ratio, CI confidence interval.

^†^ Older (age 45 years and older) vs. young adult asthma (18–45 year old).

^‡^ OR per each year increase in asthma duration.

**Fig 4:**
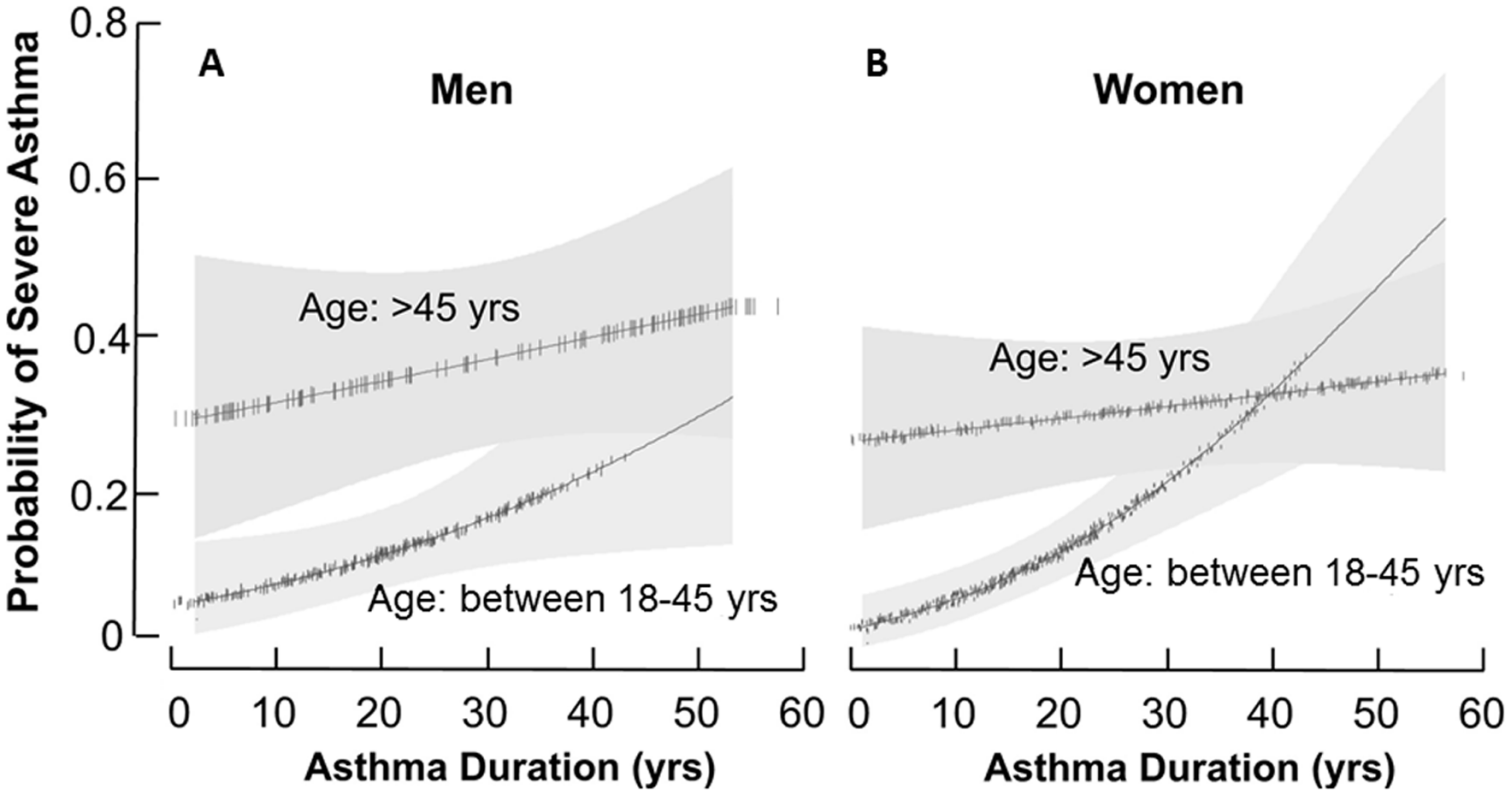
Probability of severe asthma as a function of asthma duration, stratified by gender. The probability of severe asthma in men [A] and in women [B] as a function of asthma duration stratified by age group comparing older asthma to young adult asthma. The fitted final logistic regression model, which included variables associated with severe asthma and adjusted for asthma duration, a history of Gastro-esophageal Reflux Disease (GERD) and a history of nasal corticosteroids, was used to calculate the probability of severe asthma. Young men and women have a 2% and 4%, increased risk of severe asthma per year of asthma duration, respectively. After age 45, the risk of severe asthma in women is not impacted by asthma duration, whereas risk of severe asthma in men continues to increase with increasing asthma duration.

Stratifying by gender, severe asthma was more likely in older asthmatic men [adjusted OR: 3.11(95 CI: 1.76; 5.49)], or older asthmatic women [adjusted OR: 2.52(95 CI: 1.67; 3.80)] as compared to their young adult counterparts. Severe asthma was modestly associated with asthma duration in all men [adjusted OR: 1.02(95 CI: 1.00; 1.04) per each year increase in asthma duration] “Fig 4A”. However, severe asthma was associated with asthma duration only in the younger women [OR: 1.04(95 CI: 1.02; 1.07) per year] and not in the older women [adjusted OR: 1.00 (95 CI: 0.99; 1.03) per year] “Fig 4B”. Age or asthma duration had no apparent effect on risk of severe asthma in women over age 45.

### The role of age on asthma related quality of life, medication use and healthcare utilization

Consistent with the greater probability of severe asthma, older asthmatics had more severe airflow obstruction [adjusted OR: 2.67(95CI:1.71; 4.17)], and were more likely to require treatment with high dose inhaled corticosteroids [adjusted OR: 2.28(95 CI: 1.45; 3.58)] and long acting beta agonists [adjusted OR: 1.70(95 CI: 1.11; 2.61)] “Table 3”. They also had a lower quality of life as compared to younger asthmatics [AQLQ mean ±SD: older adults 4.24±1.31, young adult 4.70 ± 1.26; mean difference (95 CI) of -0.46 (-0.62; -0.30)]. Unadjusted analysis revealed that older asthmatics expressed higher use of healthcare resources. They had a higher need for Emergency Room or Urgent Care treatment, and a higher rate of hospitalization and visits to the treating physician’s office. However, after adjustment for age related comorbidities, the use of healthcare resource was similar in younger and older asthmatics “Table 3”.

**Table 3:** Medication requirement and health care utilization with PS matching and subsequent multivariate adjustment for asthma duration and GERD.* Comparing older asthma vs. young adult asthma groups.

		Young Adult Asthma (n = 761)	Older Asthma (n = 369)	Unadjusted OR (95 CI)	Adjusted OR (95 CI)
**High dose ICS**		241 (32)	216 (60)	3.22 (2.48; 4.17)	2.28 (1.45; 3.58)
**LABAs**		418 (55)	283 (77)	2.78 (2.10; 3.69)	1.70 (1.11; 2.61)
**Asthma-related health care use—no (%)** † ‡					
	**ER or UC visit**	266 (35)	178 (48)	1.74 (1.35; 2.24)	1.1(0.75; 1.62)
	**Hospitalization**	322 (43)	209 (57)	1.76 (1.37; 2.26)	0.76(0.49; 1.17)
**Asthma-related health care use in the previous year—no. (%)** ‡					
	**More than 3 OCS burst**	151 (20)	128 (35)	2.14 (1.62; 2.83)	1.03 (0.64; 1.65)
	**Physician’s office visit**	478 (63)	290 (79)	2.13 (1.60; 2.85)	1.07 (0.65; 1.75)
	**ER or UC visit**	200 (26)	124 (34)	1.43 (1.09; 1.87)	0.65 (0.40; 1.07)
	**Hospitalization**	80 (11)	69 (19)	1.96 (1.38; 2.78)	1.08 (0.60; 1.94)

* OR denotes odds ratio, ICS inhaled corticosteroids, LABAs long acting beta agonists, ER emergency room, UC urgent care, and OCS oral corticosteroids.

^†^ History of previous health care use related to asthma.

^‡^ Health care utilization was self-reported.

## Discussion

The principal finding of this study is that aging itself, independent of asthma duration, contributes to the development of severe asthma, and that the greatest risk occurs over the time of 18 to 45 years of age. The risk of severe asthma increases by 7 percent each year until asthmatics reach age 45 years. Asthma duration is a risk factor for severe asthma in young adult men and women, supporting the concept that long-standing asthma leads to more severe asthma [24, 25]; but here, we show age is more impactful than asthma duration on risk of severe asthma. This finding is important to consideration of care of the age-related factors of severe asthmatic patients. While only 5% to 10% of asthmatics have severe asthma, this group accounts for more than 50% of the asthma related total health costs through hospital admissions, use of emergency services, and unscheduled physician visits [26–28]. The initial unadjusted analysis showed a higher healthcare utilization by the older asthmatics. Older asthmatics had more comorbidities, many of which contribute to more severe asthma. Subsequent adjustment for the majority of age related comorbidities showed that the difference in healthcare utilization was not significantly different between older and younger asthmatics. The analyses suggest that older asthmatics might have a lower level of healthcare utilization with specific attention to therapy of comorbidities that exacerbate asthma, such as gastroesophageal reflux and rhinosinus disease [29].

The findings also reveal a gender-dependent effect of age and asthma duration. Unlike men, age or asthma duration have no association to severe asthma in women over the age of 45 years. These findings are of potential clinical importance in that the cumulative effects of age and asthma duration on severe asthma risk in women are found during their young adult reproductive years. Asthma is influenced by age and sex hormones over childhood. Asthma is more prevalent in boys than in girls during childhood [30–32], but in adolescence, asthma becomes more severe and prevalent in girls [33, 34]. This shift is attributed to changes in hormonal milieu and environmental exposures that place adolescent girls at higher risk [35, 36]. In adulthood, asthma is more severe in multiparous women and during the peri-menstrual period, and its symptoms are improved by oral contraceptives in females with premenstrual asthma [35]. Asthma was more prevalent in women in the overall adult SARP cohort [37, 38], but to our knowledge this is the first suggestion of a reversal of the well-described gender switch that occurs at puberty when girls develop more asthma and more severe asthma than boys. The findings point to physiologic events in the female lung that may change at a time commonly associated with menopause. In support of this concept, the nurses’ health study reported a protective effect of menopause [39], but other studies found either no association between asthma and menopause [40, 41] or a drop of FEV1 and FVC in women with amenorrhea [42]. Hormone replacement therapy may influence asthma severity [39, 40] but was difficult to evaluate in this study because only 35 menopausal women in SARP received such therapy [43].

Irrespective of gender, the finding that risk for severe asthma is greatest and continuous from ages 18 to 45 in men and women suggests that the physiologic and structural changes, which occur over the young adult life, place individuals at increasing risk for severe asthma. Lung functions reach a maximum between the ages of 18–25, and thereafter, lung functions, in particular the FEV1, declines with age. The Copenhagen City Heart Study, which followed 17,506 subjects including 1,095 asthmatics over 15 years [44], showed a twofold decline in FEV1 in asthmatics compared with healthy individuals. However, the effect was variable among asthmatics, and neither bronchial hyperresponsiveness, nor airflow limitation correlated well with FEV1 decline rate [45]. Further, the relationship of rate of decline in airflow to the risk of severe asthma was not evaluated. Environmental factors, including occupational and tobacco exposure, are well known to influence decrements in FEV1 observed between the ages of 18 and 40 [46]. In this context, chronic inflammation and the endogenous oxidative stress related to asthma may be more robust in the younger adults, and accelerate aging of the airways *via* reactive oxygen species mediated mechanisms [47]. Although older asthmatics had higher airway and peripheral neutrophilia, functional activity of inflammatory cells decreases with aging [48]. Alternatively, the greater neutrophil numbers could be related to the aging process *per se* [49] or may indicate different underlying mechanisms of the older asthma phenotype.

One of the limitations of this study is its external validity. The SARP database was collected to study severe asthma. Most patients were recruited from asthma clinics in academic centers. Due to referral bias, their baseline characteristics may differ from the general population. Referral bias as well as the cross-sectional nature of the database could explain some of our findings including the higher trend of severe asthma in older men compared to women or even the slow increase in asthma severity after the age of 45. Additionally, the effect of age could be biased by the period and cohort effects. As asthma therapy changed over the last decades with the introduction of new comprehensive guidelines and better and more effective medications, older individuals might have received substandard therapy during their early years according to today’s standards leaving them vulnerable to lifelong uncontrolled asthma. The use of inhaled corticosteroids has been shown to slow the decline in FEV1 after initiation of therapy, but only in men, not in women [50]. This analysis may also be subject to recall bias. Although it is easier for individuals with recent onset asthma to remember their asthma duration, it may be challenging to remember the exact time of symptoms onset for those who had asthma for many years. Even taking these limitations into account, observational databases continue to be useful in making accurate predictions for individual patients [51], and the prospectively collected SARP database provides high quality data for this systematic analytic approach. Importantly, the SARP database was comprehensive, allowing us to control carefully for confounders such as corticosteroid use and hormone replacement therapy.

Altogether, this analysis reveals that aging over the young adult life is a cumulative risk for development of severe asthma, and underlies the greater incidence of severe asthma in older patients. As the proportion of the world's population over 45 years is projected to increase rapidly between 2000 and 2050 [52], investigation of the preventable age-related determinants of severe asthma, and treatment of the reversible age-related risk factors that contribute to severe asthma, will become clinically and economically vital.
